# Intimate partner violence against women in west Ethiopia: a qualitative study on attitudes, woman’s response, and suggested measures as perceived by community members

**DOI:** 10.1186/1742-4755-9-14

**Published:** 2012-08-20

**Authors:** Sileshi Garoma Abeya, Mesganaw Fantahun Afework, Alemayeh Worku Yalew

**Affiliations:** 1 Departments of Reproductive Health, Population and Nutrition, School of Public Health, Addis Ababa University, P.O. Box 9086, Addis Ababa, Ethiopia; 2 Departments of Epidemiology and Biostatistics School of Public Health, Addis Ababa University, Addis Ababa, Ethiopia

**Keywords:** Focus group discussion, Women violence, Attitude, Coping, Measures

## Abstract

**Introduction:**

Intimate partner violence against women is more prevalent in Ethiopia and among the highest in the world. This study was aimed to explore the attitudes of the community on intimate partner violence against women, the strategies women are using after the violence act, and suggested measures to stop or reduce the act in East Wollega Zone.

**Methods:**

A total of 12 focus group discussions involving 55 men and 60 women were conducted from December, 2011 to January, 2012. Discussants were purposefully selected from urban and rural settings of the study area. The analyses followed the procedure for qualitative thematic analysis.

**Results:**

Three themes (attitudes, coping strategies, and suggested measures) were emerged. Most discussants perceived, intimate partner violence is accepted in the community in circumstances of practicing extra marital sex and suspected infidelity. The majority of women are keeping silent and very few defend themselves from the violent husbands/partners. The suggested measures by the community to stop or reduce women’s violence were targeting actions at the level of individual, family, community, and society.

**Conclusion:**

In the study community, the attitude of people and traditional norms influence the acceptability for the act of intimate partner violence against women. Most victims are tolerating the incident while very few are defending themselves from the violent partners. The suggested measures for stopping or reducing women’s violence focused on provision of education for raising awareness at all levels using a variety of approaches targeting different stakeholders. It is recommended that more efforts are needed to dispel myths, misconceptions and traditional norms and beliefs of the community. There is a need for amending and enforcing the existing laws as well as formulating the new laws concerning women violence including rape. Moreover, providing professional help at all levels is essential.

## Background

In patriarchal societies which believe in male dominance the force used by a man to control his wife is seen as legitimate [1]. Moreover, studies have found that males within patriarchic societies are more violent towards their wives and children than are males in societies believing in equality [2]. Ethiopia is one of the patriarchal societies where many rural communities embrace various types of violence against women (VAW) [3]. Similarly, many rural communities in Ethiopia embrace various types of violence against women and even claim to have women who go to the point of saying: “If my husband does not beat me, it means that he does not love me,” and similar other sayings that justify violence are common [3]. Due to these facts intimate partner violence against women (IPVAW), the most common forms of VAW is highly prevalent in Ethiopia [4].

Basically, two theories, however, have heavily influenced IPVAW etiology research; social learning theory, or the idea that violence may be transmitted from one generation to the next, and the feminist theory, or the idea that male dominance in society affects interpersonal relationships [5-9]. Whatever the case, several complex and interconnected social and cultural factors are involved; all of them being manifestations of unequal power relations between men and women [8,10-13]. Indeed, the majority of researchers on IPVAW have examined and described the associated factors using socio-ecological model- the dominant one that integrates many other explanations and widely used in public health operates at the level of individual, relationship, community and society/country. Among individual level factors the use of alcohol and none or low educational/economic status are frequently cited. These factors are associated with both the perpetrators and victims of violence. At the level of the family male control of wealth, decision-making authority, habits of frequent marital conflict and significant interpersonal disparities in economic, educational and employment status are included. Community level factors include women isolation and lack of social support, community attitudes that tolerate and legitimize male violence, and poverty. At the level of society: gender roles that entrench male dominance and female subordination, inadequate laws and policies for the prevention and punishment of violence, and limited awareness and sensitivity on the part of law enforcement officials, courts and social service providers are frequently cited.

Community attitudes and actions with respect to IPVAW play an important role in shaping the social environment in which the victims are embedded. Similarly, the social environment that contribute either to condone and perpetuate or to reduce levels of IPVAW in the society plays an important role [14]. Several studies have indicated that a high risk of IPVAW in male-dominant or patriarchal societies where gender attitudes and perceptions of the community support marked inequality between men and women in addition to rigid gender roles can lead to justification and acceptance of IPVAW [15-17]. Other studies have shown that attitude towards IPVAW is one of the most prominent predictors when compared with other potential factors such as social and empowerment [18,19]. Attitudes toward infidelity by the female partner were at the other extreme: 60% of men and 87% of women believed that beating is justified if the woman was sexually unfaithful [20].

Evidence showed that women use different strategies such as tolerance, temporary or permanent separations, seeking outside help, or physical self defense from the violent husbands/partners [21]. In World Health Organizations (WHO) multi-country study on domestic violence and women’s health conducted in ten countries, for example, the victims kept silent (tolerate) and the interviewer was the first person to which many victims had ever talked about their partner’s violence [11]. Similarly, in rural Ethiopia, nearly half of the women had tolerated and didn’t talk the incident to anybody. Very few (6%) had fought back to defend themselves, and other 30% had left home on one or more occasions to escape from violent husbands/partners [3]. In addition, the WHO study confirmed that between 19% and 51% of victims had ever left home for at least one night and between 8% and 21% reported leaving 2–5 times [11].

A study on the prevalence, risk factors, and health outcomes of IPVAW on ever married/cohabited women aged 15–49 years was conducted in the study area. The magnitude and health effects of IPVAW are all-embracing. The study also identified substantial risk factors for the occurrences of IPVAW [4]. However, the communities view on IPVAW was not well explored in Ethiopian setting in general and the study area in particular. Thus, this study is aimed to explore the community attitude, strategies women are using after the violence act, and the suggested measures to stop or reduce IPVAW at the study area.

## Methods

The study was conducted in one urban and four rural districts in East Wollega Zone, which is one of the 18 administrative zones of Oromiya National Regional State. East Wollega Zone is located at the western part of the country 331 km far from Addis Ababa, Ethiopia. The 2011/12 projected total population of East Wollega zone is 1,610,816 out of which 789, 299 (49%) are males and the rest 821, 516 (51%) are females [22]. Different ethnic groups, such as Oromo, Amhara, Gurage and Tigre are residents of the zone, out of which 85% of the dwellers were Oromo and ‘*Afan Oromo*’ is the official and working language [22].

Qualitative (FGD) data was collected between December, 2011 and January, 2012. The number of focus group discussion was determined by saturation of ideas - until a point where no more new ideas emerged. A total of 12 focus group discussions comprising of 115 discussants (55 male and 60 female) was conducted. Four groups were from urban (two groups of males and two of females) and eight groups were from rural residents (four groups of males and four of females). The discussants were employees from different sectors, religious and community leaders, elders and residents of the study area. Each group consisted of 8–12 participants. The discussants were selected with criteria of being ever married/cohabited, having not been involved in the previous survey [4] and being not related with each other. They were selected using purposive sampling method with the support of the local leaders and health care professionals as they were well familiar with the conditions of the area. The FGDs were categorized by sex, area of residence and employment statuses to capture heterogeneity among different subgroups and to allow for homogeneity within a group [23].

Open-ended unstructured and flexible discussion guide was prepared based on the objective of the study. The discussion guide for the factors involved in the occurrences of intimate partner violence against women was prepared based on the ecological model [8] of understanding IPVAW. During data collection and initial analyses, this pre-understanding was put within brackets [24].

Two trained female research assistants (a social worker and an anthropologist) moderated the female group activities while the principal investigator and male anthropologist guided the male groups. One female and one male assistant from the team organized the FGDs and handled tape recordings. The discussions were tape-recorded and all observations made were recorded as field notes. The discussions were conducted in a quiet place to encourage free discussion without any threats and each lasted for 60–90 minutes.

The recorded FGDs were transcribed verbatim in the regional language (*Afan Oromo*). Later, it was translated into English by the principal investigator together with the moderators. To assure the validity of the translation, another person, proficient in both languages checked and commented on it so as to incorporate changes into the report. Many of the post-FGD questions and interactions were not recorded to include into the analysis. Following the steps of qualitative thematic content analysis [25-27], the texts were imported into the Open Code 2007 program to facilitate the coding process [28]. After reading the transcripts, the researchers performed open coding of the texts, constantly comparing similarities and differences by going back to the original text. In the next step, selective coding was performed and relevant codes were further conceptualized leading to the development of categories. Several categories were taken together to form themes. During the coding process, relevant quotations from transcripts were put into memos and incorporated to illustrate main ideas during the write-up. Finally, understanding of multiple forms of information collected and triangulation of different data sources was made to verify the findings.

The research proposal was examined and screened for scientific and ethical integrity by the institution’s review board (IRB), the highest body for approving research in the College of Health Sciences, Addis Ababa University. To this effect, a letter with green light to carry out the study was issued and an official letter was written to the regional and local authorities by the school of public health. Formal permission and verbal consent was secured from administrative officials at different levels of governmental Authorities and the local community leaders. Furthermore, the ethical guidelines of VAW research was strictly followed [4]. Informed verbal consent was obtained in advance from the respective FG discussants to record their responses. They were also told to switch-off the audiotape, if they feel that there are issues which should not be recorded. The researcher ensured that all collected information is kept and used in such a manner that confidentiality, anonymity and privacy of all participants are maintained, bearing in mind the sensitivity of the topic.

Multidisciplinary research team, verbatim transcriptions, and predefined analytical procedures were used to promote the study rigor. During the analysis the fitness and relevance of emerging categories to the research question were tested by constant comparison and checking between the text, codes and categories. Moreover the Lincoln and Guba’s model [29] of trustworthiness has been applied. Credibility was assured by continuous interaction with discussants in the study area. Follow-up interviews with participants were conducted and member-checking was done to verify the findings. Triangulation of data collection methods was done as field notes were also used to collect data. Transferability was enhanced by purposive sampling of the discussants. Detailed and thick/dense description of results as well as literature control was done to support the findings. Confirmability was ensured through the use of an independent coder who analyzed the data independently and a consensus discussion was held to agree on adopted themes and categories [30].

## Results and discussion

### Results

The number of focus group discussion was determined by saturation of ideas- until a point where no more new ideas emerged. The saturation point of information has been reached after the twelfth discussions. A total of 115 discussants (55 men and 60 women) participated into the discussions. The median age of the discussants is 40.4 years (range: 23 to 78 years). Details of the socio-demographic characteristics of the discussants are shown in Table1.

**Table 1:** Socio-demographic characteristics of the discussants, East Wollega zone, December, 2011 to January, 2012 (n = 115)

**Characteristics**	**Categories**	**Frequency (Percent)**
Place of residence	Urban	34 (30)
	Rural	81 (70)
Sex	Male	55 (48)
	Female	60 (52)
Educational status	Illiterate	8 (6)
	Read and write	4 (4)
	Primary education	19 (17)
	Secondary education	36 (31)
	Tertiary education	48 (42)
Occupation	Employee	48 (42)
	Religious leader	18 (16)
	Local elderly	25 (21)
	Adults (community residents)	24 (21)

NB- Employees were from the offices of administration, health, education, police, prosecutor, court, women’s affairs, social affairs, and non-governmental organization.

The main themes described during the analysis were: description of violence that occurs to women from their husbands/partners, explanation on the possible factors leading to women’s violence and its consequences, community attitudes on IPVAW, women’s response after the attack, suggested intervention area, the currently available and functional social and/or legal services at the area, and the current statuses of IPVAW at the study area. However the main concern of this paper is the analysis of the views of the community on attitudes on IPVAW, women’s response after the attack, and the suggested measures to stop or reduce IPVAW**.** These themes are described by categories, sub-categories, and codes (Table2).

**Table 2:** Descriptions given for intimate partner violence against women by themes, sub-categories and selected codes, East Wollega Zone, Western Ethiopia, December 2011 to January 2012

**Themes**	**Sub-category**	**Codes**
Attitudes towards IPVAW	Accepting IPVAW	Approve, tradition, tolerance of women, community norms, controlling
	Not accepting IPVAW	Hair split argument, give advice, sanction, exclude from *Edir*, no support
Woman’s reaction	Tolerate	keeping silent, silently live, weep and live, feeling shame
	Seek help	Tell, Women’s Affairs, elders, police, *kebele* leaders, courts, relatives, neighbours
	Leave home	Separate temporarily or permanently, divorce, disappear
	Self defense	Fight back, beat him, use force, disregard
Suggested measures at various levels	Individual level	Create awareness on education, economic issue, females right
	Family level	Hold discussions on family issue, understand each other
	Community level	Change attitudes and beliefs through mass education, school and religious activities
	Society level	Raise awareness on legal codes and related services, gender roles, advocacy

### Attitudes of the Community towards Intimate Partner Violence against Women

The traditional tendency that places women in a subordinate position to men has led to a culture of justification of accepting IPVAW. This act is often justified by men as a form of control of the family. However, from these FGDs two divergent ideas emerged on the attitudes towards women violence inflicted from their intimate partners.

### Conditions under which the Act of IPVAW Could Be Acceptable

A.  **Failure to give birth and suspicion for infidelity**

 The acts of IPVAW could be acceptable by the community if the woman commits extra-marital sex, is suspected for sexual infidelity (adultery), is always in dispute with her husband, her neighbors or community members, does not obey the husband/partner, or fails to give birth under all conditions. 

" *“…most men cite a paragraph in the bible states the wife has to respect her husband. They are repeatedly using this saying when they want to attack their wife/partners in all circumstances like suspecting her for infidelity”* (A 37 years religious leader from female group)."

" *The society pushes families to have more children even if the woman resists. Commonly people say “garaan haadhaa qalqalloodha,” which literally means, “mothers’ womb is like a container”* (A 34 year-old woman from prosecutor’s office)."

B.  **Behaving as controlling and non-domestic women**

 The community believes in women accusation when they attempt to speak for their own rights. Most people also stigmatize such women labeling them as deviant, big-headed, and uncultured. The community also attaches connotative expressions like non-domestic to such women when they actively participate in public affairs. For example, both men and women groups noted that people in their community accept wife beating as it is the appropriate way to correct the women.

" *There is a saying, “uleen suphee duwwaa cabsa” to mean, “A stick breaks only a clay pot.” Mostly elders in this community believe in the importance of stick for controlling women. Such a traditional belief targets the way a woman dialogues with her husband. If a woman swears at her husband, it is considered as looking down on him, so he has to punish her* (A 41 year-old man from a rural community)."

### Conditions under which the Act of IPVAW Could not Be Acceptable

A.  **Hair splitting arguments**

 According to some of the participants, currently there is some degree of change in the perception of the community on IPVAW. The community is taking different measures against men who frequently attack their wives or who exhibit a violent behavior. The majority of the participants from both groups expressed that IPVAW is an intolerable act, especially if the man commits adultery, is always intoxicated, and always raises hair split arguments like for instance the dinner is not delicious and the like.

" *Most members of our society know that attacking women is a crime. Usually, if the man raises hair splitting arguments and is always in dispute with his partner, the act is considered as intolerable. Even if there could be a disagreement between a husband and a wife, it should not lead to abuse and has to be solved through discussions* (A 34 year-old woman from the Education Bureau)."

B.  **Exclusion from social support system**

 The majority of participants from the female groups extensively discussed the protection of the right of women as they are part of the society. Others also mentioned that if a husband keeps attacking his wife on trivial issues and always quarrels with other people in the community, he is excluded from the local social support system (edir) in the form of sanctions.

" *…. Despite the traditional roles of men as head of the family, the community considers women as daughter, sister, wife and mother. For this reason, we do not accept attack under any circumstances. Occasionally, if the husband keeps attacking his wife on trivial issues and always quarrels with other people in the neighborhood, the community rejects his act and excludes him from social affairs like edir. However, to do so, it should be ensured that the woman has fulfilled the expectations of the husband/partner and the community, like for example, obeying the husband, giving care for the children, giving birth, and not having extra-marital sexual relations”* (A 46 year-old man, rural education office)."

### Women’s Reactions to Intimate Partner Violence (Coping Strategies)

According to the majority of the participants, most women do not report cases of violence by their intimate partner primarily due to the fact that they may be stigmatized and ashamed by the community. In some cases, it may result in life of discrimination with consequences like difficulty in re-marriage. Basically, four sub-themes emerged on strategies that women use when such an incident occurs in the study area.

A.  **Tolerance**

 Most discussants mentioned that their culture dictates women not to go back to their parents or elsewhere once they got married for they are expected to keep the family net intact, especially for the welfare of their children. Thus they tend to stay tolerant of every challenge from their abusive partner. 

" *…. In the beginning, I used to cry and lock myself up in a room. But now, I gave up, and I would not leave my husband. I can’t stay away from my kids or make them suffer without a father figure. They are my life, and I would do anything for them….” This is the reason why I stay on tolerating violence by my husband* (A 33 year-old woman from rural community)."

" *I myself passed so many nights in my compound under banana and went back to home in the morning for the sake of my children* (a 27 years female from Nekemte town)."

 Furthermore, most women in the study area have no awareness about their rights and prefer to remain tolerant of everything from the husbands/partners. Some also said that the traditional norms of the society discourage females not to discuss their problems with others. In addition, most women live with the principle of “*obsuu”* and “*dhoksuu*” which means “*remaining patient”* and *“keeping secret*.” This means, females should tolerate and hide the challenges they face from their partners. 

" *The tradition itself expects females to be a tolerant wife. That is why “dandeessuu,” meaning someone with high degree of endurance, is a very common bride’s name given by the family of the husband at marriage* (A 35 year-old man from the Education Bureau)."

" *“yartuun fira hamattii baddubatuun dhirsa hamatti” literally to mean “bad wife backbite her relatives while hopeless wife backbite her husband.”* (A 36 years old woman from urban)."

The woman as a victim accepts that there is nothing she can do about the abuse. She accepts that she was just unfortunate to marry an abusive partner but she is prepared to endure the relationship. A 52 years religious man from rural community noted, *“…the woman has made a commitment to their marriage until death. Therefore, she will not go away”.*

B.  **Leaving home**

 The discussants from both groups mentioned that most women in the study area, mainly those in the rural areas, leave home either temporarily or permanently when they feel they cannot bear the violence any longer. In fact, because of culture and traditional norms of the society, men are not expected to leave the house. Moreover, court personnel remarked that most of the time women face severe attacks from their husbands and leave home with no claim of any property in their home. 

" “*I was married at age of 15 and lasted for 22 years in marriage. In this stay I left my home and went back to my parents for about 20 times. My mother said me “what do the community says about us when you come back to us from your husband. So you have to go to him.” She sends me back when elders come without talking about the problems I encountered there* (A 37 years from urban)."

" *In my vicinity, a husband and a wife were always in dispute during the night time. She shouted every time he came drunk and started annoying her. She always went to his relatives and came back after things cooled down. But one day, he severely attacked her on the back and she was admitted to a hospital. After she recovered, she permanently left home and went back to her family with no appeal to the legal system* (A 61 year-old man from the urban)."

 The court personnel extended their idea that most of the time women face severs attack from their husbands and leave home without giving care for their property in their home. In this regard, when both partners come to the court system and the woman win, the husband sends local elders for mediation and she lifts her appeal with the pressure.

C.  **Self-defence**

 Few women in the area resort to physical confrontations to have their rights respected and protect themselves from harsh physical or verbal attack. This was reflected in the discussions among urban respondents.

" *…. Let me share you my personal experience. After long time tolerance and weeping, I tried to beat him to defend myself when he severely attacked me on the stomach* (A 29 year-old house wife, urban)."

 A religious leader from the rural community also said:

" *…. I have seen a woman in my locality that goes to the extent of injuring his genital organ with knife after several disputes. The woman took this measure for self-defense because the man has become addicted to violent behaviour* (A 57 years religious leader from rural community)."

D.  **Seeking help/telling others**

 Most victims, as a first measure, usually go to the local/village elders, relatives, or close friends for arbitration. In fact, these people often advise the couple to tolerate each other. But if the case is not settled, they resort to more formal structures such as the *kebele* social court, women’s affairs, police or court system. Even then, there are a number of cases where these women are not taken serious because of influence from long standing traditions of the community. The cases below attest to such practices.

 A judge from the rural area expressed his experience on a case where a woman sued her husband for beating her. There, the judge asked the man, “Why did you beat your wife violating her right?” Then the husband replied, “*Respected judge, I did not beat her on the right, but on her left side.”*

 In this regard, the women groups draw attention to the fact that females are excluded from the local elders for arbitration of disputes between couples in their community. For instance one of the female group discussants said,

" *.… One day, I went to take part in the local elders group to mediate disputed couples. In the group there were seven males and I was the only female. I was unfortunately late to arrive and they were waiting for me. In fact, they did not know me and were expecting the eighth male. But when I arrived, they were surprised and angrily remarked, “We wasted our time waiting a woman”* (A 46 year-old woman from the urban)."

 Most participants extensively claimed that women are not allowed to be members of an arbitration group. This implies that decisions made usually favour males. The mediators mostly pressurize females to accept their decision no matter how much she was victimized. Moreover, how women are treated by the police officers was an issue of lively discussion among female participants.

" *“A woman went to the police office to sue her husband. The investigator was a policeman who had three co-habitants. He sent the woman to local elders advising her not to waste her time. He said, “Abbaa manaaf haadha manaa sireetu araarsa” literally meaning, the bed mediates between a husband and a wife. This is a problem. The problem is not the issue of policy but its implementations” *(A 34 year-old woman from Women’s Affairs Office)."

 Since women are not allowed to be parts of arbitrators, the decisions are usually biased towards males. They usually pressurize females to accept their decision no matter how she is victim of the conflict. Members of the community use proverb which encourage females’ inferiority. For instance,

" “*dubartiin dheertuu malee beektu hinqabdu” which means; “we do not find wise women but tall”*(a 32 years female from urban residents)."

### Community Suggested Measures

The other theme emerged was community’s perception towards measures taken to stop IPVAW. Other sub-categories were observed after selective coding of measures to be taken at the levels of individual, family, community, and society was made.

A.  **At an individual level**

 In most discussions, the first measure should focus on intensive provision of formal and informal education for females on their legal rights and problems of cohabitation. Moreover, the majority of the participants suggested that the concerned bodies (religious institutions and municipality) should provide premarital, sexual and family life education.

" *…women have to be advised on not simply co-habiting with a man without thorough scrutiny of him as this problem is pervasive in the area. Pre-marital education should be provided by the concerned bodies like the municipality and religious leaders”* (A 39 year-old woman from the local court)."

B.  **At family level**

 Advising the couple to discuss all family issues in a gentle and courteous way was briefly raised in all groups. They emphasized that a husband and a wife should have respect for each other from the very beginning, because once a dispute occurred between the couple, it becomes difficult to manage. They also stressed the importance of teaching the couple about their income, family problems and the need for one of the couple to try to calm the other down when she becomes temperamental. They said a family issue should be discussed and solved within the family.

" *Since family is a base for the community and eventually for the country, solving a family problem has to be the responsibility of all citizens. Tolerance is the major mechanism of reducing violence between couples. As an Oromo proverb goes, “obsaan aannan goromsaa dhuga” meaning, “Someone tolerant drinks the milk of the heifer” (implying this is the best milk) to indicate the importance of tolerance in the family. Behaving this way solves problems related with alcoholism and temperament* (A 49 year-old male community leader)."

C.  **At community level**

 Most discussants stressed the importance of formal social support like government assistance programs and criminal justice, and informal assistance by the local elders, friends, relatives and others.

" *According to the custom of the area, before any further step, elders look into the dispute between couples for more than three times. If they fail to resolve the problem, then the case is taken to the court. However, because the custom is highly valued, the court could even send it back to the local elders before making a legal decision. This is the procedure we follow. In the community, elders are responsible for thoroughly investigating cases and giving advice to the wrongdoer and mediate the couple* (A 45 year–old male from Prosecutor’s Office)."

To stop or reduce IPVAW, it is prudent to educate the whole community. Changing the attitudes of the society regarding the rights of the women is another working area for everybody in the community. If couple have thought sitting together, they can understand for each other and improve their behavior; they can openly discuss every issue and hence reduce the chance of getting dispute. Also it is better to use religious institutions, local social support system like edir, schools and other relevant places to teach the society. 

" *The best solution is increasing the community’s awareness using media and forums. In addition, schools should teach about gender issues from the very beginning so that the generation can be changed* (A 78 years local community elder)."

" *Some community members are against the act of violence and still there are some individuals who can give approval for such acts. To balance the community responses, awareness creation and awareness rising should be given on gender issues through media and religious institutions on continuous bases* (A 42 years male from justice office)."

All groups emphasized the importance of collaboration among different organizations for stopping or reducing IPVAW. The behavioral change strategy should be focusing on everyone in the community.

" *….last week a man injured an eye of a woman who owns a grocery. She came to our office for help and we directed her to the police. But a group followed and warned her saying, “If you withdraw your appeal, he will pay you compensation, but if you proceed, you will lose all your customers.” She accepted the second option. I personally met one of the members of the group mediating the case and asked him why they interfered. He replied, “Harree fi dubartiin duruu ni reebamti, maal ho’ista?” which means, “It is a tradition for the women and donkeys to be beaten, so why do you exaggerate****?****”* (A 47 year old woman from an NGO)."

 Particularly the rural discussants gave emphasis for providing equal services for all people in the community irrespective of their economical status and ethnicity.

" *“…when rich and well to do families are in dispute, the community interfere and tries to mediate them as early as possible. But when poor family like me is in dispute and females are in problem no one interferes and tries to mediate. In addition, poor families cannot be a member of local social support (edir) as they do not afford the monthly contribution. Basically such social supports is formed by members of the local villages and lead by elders for helping people in all aspects and interfere and/or mediate the family dispute* (A 40 years daily laborer female)."

D.  **At society/country level**

 Some participants in this study pointed out that much is expected of all organizations in general and the implementing agencies in particular. It is important to hire experts on family and marriage issues equipped with knowledge of family mediation. Others also suggested that the government should amend or codify acceptable evidences in investigating violent acts because many people complain that the criminal code of Ethiopia demands eyewitness from three people and medical certificate from health institutions to decide that the offender is guilty. For instance, in cases of rape, it is impossible to have eyewitness as the act takes place in a hidden environment. 

" *There is a gap between policy on the paper and its implementations. For instance if we take the so called women’s’ associations, they are not properly working. Even at some places they do not have sufficient knowledge on the issue itself. As a result they are not successful in mobilizing women to attend different awareness rising forums. On the other hand, the legal bodies ask eye witness for serious criminal act like rape* (A 47 years old male from education office)."

" *The law of the land and the police demand that the victim produce evidences like witnesses from at least three people and medical certificate from health institutions. This is not right. What is expected of the victim should be reporting the case. Investigating the issue should be the job of the police, because such attacks take place in secret so that the victim cannot produce evidence***(**A 29 year-old women from the Education Bureau)."

 In addition, they recommended that concerned bodies like the police officers, experts in women’s affairs, the prosecutor and judges be equipped with appropriate knowledge and skills necessary to handle such cases. Additionally, there should be legal protection for the victim so that the offender does not attack again or revenge. Majority of the participants suggested that the court establish a special center that works on GBV and emphasize on empowering women.

" *When we think about the solution, we have to think about who is responsible. Who should be involved? As the problem is complex, it needs the involvement of different stakeholders. Concerned bodies should work to practically implement the gender policy of the country and draft new laws or amend the existing ones to make them appropriate to deal with violence against women* (A 36 year-old female from Social Affairs Office)."

## Discussions

The first theme explains the divergent idea of the community towards IPVAW. The sub-categories for the second theme on women response includes tolerance, temporary or permanent separation, seeking outside help, and physical/verbal self defenses. Thirdly, the theme on the measures to be taken to stop or reduce IPVAW was focusing the activities at individual, family, community, and country levels.

The majority of the discussants have mixed and divergent feelings to express the community attitudes on IPVAW. Some justified the accusation of women by their husbands if she committed extra marital sexual affairs. This goes with the finding from other similar study justified wife beating triggered by an action that is socially unacceptable like sexual infidelity [31]. Similarly the EDHS of 2005 indicated that the majority of women believed for a husband to be justified in beating his wife at least for one reason if she is: not completing housework on time, refused sex, disobeying her husband, being unfaithful or questioning him about his extra-marital relationships [32]. These all implies the traditional norms place women in a subordinate position to men and have led to a culture of justification of IPVAW. However, some of the discussants were claiming the act of wife abuse to be a criminal act because currently it is given attention by the government.

The majority of the discussants assured the victims in their community are living with their abusive partners tolerating the incident. This could be justified as lack of awareness of the women about their rights. Moreover, the influence of traditional believes and norms in their area are more pronounced. This is similar with the findings from Spain that showed high level of women violence tolerance was associated with low awareness about their rights [14]. The reason why abused women hide violent attacks to their children, family, friends, and neighborhood is to avoid outside interference and later reprisal from the abuser which forces the victim to prefers and live with the abusive partner silently. Women often considered it as a shameful to share such personal problems with others [33]. For example, previous study from rural Ethiopia showed nearly half of the women kept silent after the violent attack from their intimate partners [3]. This explains why many women do not discuss violence with anyone else that indicates either they consider it normal feature of life or ashamed of revealing the violence.

In most cases the victim primarily tries to report the incident to their relatives, close friends, or local elders for advice and support. But these depend on the severity of the violent act that ranges from verbal threats to severe physical aggression. This finding goes with the result from Nicaragua which indicated that the woman’s decision to seek help may be triggered by specific incidents like when attacked severely, affecting the children welfare, and destroying properties [21].

In the study area few women, especially from urban are fighting against their abusive husbands/partners either physically or verbally in order to protect themselves. This is in contrast with finding from Nepal and Nicaragua where the majority of the women reported in defending themselves from abusive partners [21,34]. However, it is corroborating the finding from rural Ethiopian showed only six percent of the victims had fought back to defend themselves from the violent husbands/partners [3]. In Ethiopian context, it is evident that fighting back to the husbands is not culturally accepted and thus it is shameful for the woman to hit her husband though, this by itself could be one strategy in minimizing damage from abusive partners.

Some women mainly from the rural go to their relative house and to a lesser extent to friends or neighbors. This goes with the study result from Butajira, Ethiopia indicated about one in three women left their home either temporarily or permanently because of the incident [3]. Actually some culture specific factors that are related with the practice of women leaving home or staying somewhere without their partner can determine this strategy. This depends on the availability of places of safety for women and their children. However, ending a relationship does not necessarily reduce a woman’s risk, as some partners become even more violent when women leave or attempt to leave the relationships [35].

The suggested measures for stopping or reducing IPVAW are targeting the factors described in the ecological model for the occurrences of IPVAW at the levels of individual, relationships/family, community, and society/country [8]. This is because of the fact that IPVAW is a multifaceted issue with psychological, social and environmental roots [36]. According to their suggestions the measures require a variety of approaches targeted different stakeholders, including government and nongovernmental agencies, law enforcement agencies, the health and education sectors, the line ministries such as the women affairs, and other faith based organizations (FBOs). Their suggestions are in line with the recommendations from other similar studies from around the world [3,21,31,37].

Currently there are several institutions which deal specifically with Gender Based Violence (GBV), including Women’s Affairs Offices (federal, regional, district (the lower administrative unit) and in some cases *kebele (*the lowest administrative unit in Government structure) level), the judiciary system (police, prosecutors office and court), health institutions, Ethiopian Women’s Lawyers Association (EWLA), women’s associations and schools. However, the effectiveness of these institutions to prevent intimate partner violence against women, mitigate its impact and enforce the laws has been reduced due to a number of factors, including limited capacity of the institutions, attitudes of the personnel, accessibility and affordability of the services including the quality of services provided to the community and lack of knowledge by women about the existence of such institutions. The community suggested measures also support these notions.

As to the strengths of this study, we have used a multi-dispensary research team. Moreover, the methodological efforts to achieve diverse and representative sample of discussants from the community and the rigor of the coding and analysis phases are meticulously applied. We also think that the discussions were open and free. However, concerning the limitation, as any other qualitative researches the study results may not be generalized to all other areas of the country. Despite this, we believe that this study has contributed a deeper understanding and knowledge for people in this field and similar area.

## Conclusion

The study found that the majority of the discussants stated as the victim uses silence or tolerating the violent husbands/partners. This is clearly due to the traditional norms and attitudes of the community towards IPVAW. The study also showed, very few victims can defend themselves from the violent husbands/partners. Biased arbitration is marked excluding women from elders in the arbitration or mediation system. The penal code of the country has a loophole for supporting the rape cases that requests permissible or tangible evidences, since the act of rape is always committed secretly or behind closed door. It is recommended that more efforts are needed to dispel myths, misconceptions and beliefs that condone IPVAW. For mitigating IPVAW influencing personal relationships and creating healthy family environment is needed by all stake holders. Providing professional help and support at all levels as well as for the families who are already experiencing the act is also crucial. There is a need for amending and enforcing the existing laws as well as formulating the new laws concerning rape.

## Competing interests

The authors declare that they have no competing interests.

## Authors' contributions

All three authors were responsible for the design and conduct of the study. Coding and interpretation of findings and drafting of the manuscript were done by the three authors. The authors read and approved the final content of the manuscript.

## Authors' information

GS is working as lectures in departments of Reproductive Health, Population and Nutrition, School of Public Health, Addis Ababa University, P.O. Box 9086 Addis Ababa, Ethiopia. FM is a professor of Public Health working in departments of Reproductive Health, Population and Nutrition, School of Public Health, Addis Ababa University, Addis Ababa, Ethiopia. WA is a statistician working in departments of Epidemiology and Biostatistics, School of Public Health, Addis Ababa University, Addis Ababa, Ethiopia.
